# Role of the Emphysema Index Combined with the Chronic Obstructive Pulmonary Disease Assessment Test Score in the Evaluation of Chronic Obstructive Pulmonary Disease

**DOI:** 10.1155/2021/9996305

**Published:** 2021-10-13

**Authors:** Qi Ding, Xia Wei, Jie Li, Yan-Zhong Gao, Shu-Di Xu, Nan Yu, Jiu-Yun Mi, Bai-Bing Mi, You-Min Guo

**Affiliations:** ^1^Department of Pulmonary and Critical Care Medicine, The Ninth Hospital of Xi'an Affiliated with Xi'an Jiaotong University, Xi'an, Shaanxi 710054, China; ^2^Department of Radiology, The Ninth Hospital of Xi'an Affiliated with Xi'an Jiaotong University, Xi'an, Shaanxi 710054, China; ^3^Department of Radiology, The Affiliated Hospital with Shaanxi University of Traditional Chinese Medicine, Xianyang, Shaanxi 712046, China; ^4^Department of Epidemiology and Biostatistics School of Public Health Xi'an Jiaotong University Health Science Centre, Xi'an, Shaanxi 710061, China; ^5^Department of Radiology, The First Affiliated Hospital of Xi'an Jiaotong University, Xi'an, Shaanxi 710061, China

## Abstract

**Background:**

This study aimed to evaluate the efficacy of the emphysema index (EI) in distinguishing chronic bronchitis (CB) from chronic obstructive pulmonary disease (COPD) and its role, combined with the COPD Assessment Test (CAT) score, in the evaluation of COPD.

**Methods:**

A total of 92 patients with CB and 277 patients with COPD were enrolled in this study. Receiver operating characteristic (ROC) curves were analyzed to evaluate whether the EI can preliminarily distinguish chronic bronchitis from COPD. Considering the heterogeneity of COPD, there might be missed diagnosis of some patients with bronchitis type when differentiating COPD patients only by EI. Therefore, patients with COPD were classified according to the CAT score and EI into four groups: Group 1 (EI < 16%, CAT < 10), Group 2 (EI < 16%, CAT ≥ 10), Group 3 (EI ≥ 16%, CAT < 10), and Group 4 (EI ≥ 16%, CAT ≥ 10). The records of pulmonary function and quantitative computed tomography findings were retrospectively analyzed.

**Results:**

ROC curve analysis showed that EI = 16.2% was the cutoff value for distinguishing COPD from CB. Groups 1 and 2 exhibited significantly higher maximal voluntary ventilation (MVV) percent predicted (pred), forced expiratory volume in 1 second (FEV1)/forced vital capacity (FVC), maximal midexpiratory flow of 25–75% pred, carbon monoxide-diffusing capacity (DLCO)/alveolar ventilation (VA), FEV1 % pred (*p* ≤ 0.013), and maximal expiratory flow 50% pred (all *p* < 0.05) than Group 4. FEV1/FVC and DLCO/VA were significantly lower in Group 3 than in Group 2 (*p*=0.002 and *p* < 0.001, respectively). The residual volume/total lung capacity was higher in Group 3 than in Groups 1 and 2 (*p* < 0.05).

**Conclusions:**

The combination of EI and CAT was effective in the evaluation of COPD.

## 1. Introduction

Chronic obstructive pulmonary disease (COPD), a heterogeneous chronic inflammatory airway condition, is one of the leading causes of death with increasing morbidity and mortality worldwide [1, 2]. The definition of COPD is given as a common, preventable, and treatable disease that is characterized by persistent respiratory symptoms and airflow limitation that is due to airway and/or alveolar abnormalities, usually caused by significant exposure to noxious particles or gases [3]. The diagnosis and assessment of COPD were mainly based on the pulmonary function according to the Global Initiative for Chronic Obstructive Lung Disease guidelines [4]. In 2011, the symptoms and history of acute exacerbation were also included in the COPD evaluation system [5]. In addition, the St George's Respiratory Questionnaire (SGRQ) has been widely used for measuring the health status of COPD patients [6].

The COPD Assessment Test (CAT) score was recently developed for health status measurements based on the SGRQ [7], which evaluates the impact of symptoms on COPD patients [8]. A CAT score ≥10 is recommended as the threshold for severe symptoms in COPD patients [4]. The CAT is a simple tool comprising eight questions and can distinguish between responses to pulmonary rehabilitation. The CAT score is significantly better in patients with stable COPD than in those with exacerbations [9], and it is negatively correlated with the percent forced expiratory volume in 1 second (FEV1%) [9, 10]. The above assessment approach has certain merits and is more applicable in clinical practice but lacks sufficient objectiveness to reflect the pathological features of COPD patients. Identifying novel indicators is necessary to improve the evaluation system. In addition, emphysema is an important COPD phenotype, which is defined as a condition of the lung characterized by abnormal, permanent enlargement of airspaces distal to the terminal bronchioles, accompanied by the destruction of their walls, and without obvious fibrosis [11]. Quantitative computed tomography (QCT) can be used to quantitatively assess lung structure and function of patients with emphysema [12]. QCT can be used to discriminate the subtypes of emphysema-dominant and airway-dominant phenotypes of COPD and predict the prognosis of COPD patients [13, 14]. Computerized tomography (CT) emphysema index (EI) has been recently reported to be useful in predicting forced expiratory volume in 1 second (FEV1)/forced vital capacity (FVC) [15]. The low attenuation area (LAA) can be applied to calculate the EI in order to assess the extent of emphysema, which is beneficial for COPD management [16, 17]. Besides, COPD is a very heterogeneous disease, with chronic bronchitis (CB) at one end and emphysema at the other end in the typical COPD spectrum, and most patients have some characteristics of both [18]. CB is defined as chronic cough and sputum production for at least 3 months per year for two consecutive years [18, 19]. CB is often the precursor for COPD, which had been reported to increase the severity of the disease (aggravated exacerbations and respiratory mortality) in COPD patients and was considered as a COPD phenotype [20, 21]. Whether the EI can be used to distinguish CB from COPD or to diagnose COPD remains unclear.

Currently, combining the CAT with EI for the evaluation of COPD has not been evaluated [22]. Considering that it is not sufficient to evaluate structural lung changes according to the pulmonary function, other methods to explain the observed heterogeneity and assess COPD are required [23]. Thus, in this study, we attempted to examine the diagnostic efficacy of EI to differentiate COPD from CB. We also evaluated the effects of combining the CAT score with EI to stratify the subgroups of COPD patients.

### 1.1. Quick Look

#### 1.1.1. Current Knowledge

The current assessment approach lacks sufficient objectiveness to reflect the pathological features of COPD patients. Identifying novel indicators is necessary to improve the evaluation system. The emphysema index (EI) has been reported to be useful in predicting forced expiratory volume in 1 second (FEV1)/forced vital capacity (FVC). Combining the CAT with EI for the evaluation of COPD has not been evaluated.

#### 1.1.2. Contribution of This Paper to Our Knowledge

The combination of EI and CAT score was effective in distinguishing COPD patients of different phenotypes. The emphysema-dominant patients had a poorer pulmonary function. The non-emphysema-dominant patients benefited from the symptom assessment. Our findings may be helpful in guiding individualized therapy and the clinical management of different subtypes of COPD.

## 2. Methods

### 2.1. Subjects

Patients with respiratory diseases, including those with COPD and CB, who were admitted to our hospital between 2014 and 2017, were enrolled in this study. COPD was diagnosed based on an FEV1/FVC of <70% after bronchodilator use [24]. The symptom severity of COPD patients was evaluated based on the extent of emphysema according to the EI and CAT score. CB was determined by the presence of chronic cough and sputum for ≥3 months per year, persisting for ≥2 consecutive years [25]. Patients with an FEV1/FVC ≥70% were classified into the CB group.

The exclusion criteria included (I) patients aged <40 years; (II) patients who were pregnant; and (III) patients with serious comorbidities of the respiratory system and who had previously undergone surgical treatment, or were unable to complete the pulmonary function test. Records of patient characteristics including age, sex, body mass index, smoking status, blood routine, arterial blood gas, and modified Medical Research Council dyspnea index were collected for further analysis.

This study was approved by the Ethics Committee of the Ninth Hospital of Xi'an Affiliated with Xi'an Jiaotong University (Approval No. 2014001). Written informed consent was obtained from the patients.

### 2.2. COPD Assessment Test

The CAT score was developed to provide a simple and reliable measure for the impact of COPD on health status measurement [26, 27]. The CAT has eight items covering aspects such as symptom, energy, sleep, and activity. Each item can be scored from 0 to 5, and the total score ranges from 0 to 40. The impact of symptoms for patients were classified as slight impact with total score <10 and high impact with a total score ≥10.

### 2.3. Pulmonary Function Test

Spirometry, pulmonary diffusion function, and bronchial diastolic function (Jaeger MasterScreen, CareFusion Germany 234 GmbH, Baesweiler, Germany) were evaluated following the American Thoracic Society and European Respiratory Society recommendations [16]. The FEV1% predicted (pred), FEV1/FVC, FVC, maximal expiratory flow (MEF) 25% pred, MEF 50% pred, and maximal midexpiratory flow (MMEF) 25–75% pred were measured by spirometry, whereas pulmonary diffusion function and residual volume (RV)/total lung capacity (TLC) were measured using the single-breath method. All data were recorded after the administration of an inhaled bronchodilator (salbutamol 200 *μ*g).

### 2.4. CT Scan

Imaging was performed using a 64-slice QCT scanner (Somatom Definition AS, Siemens, Erlangen, Germany) with patients in the supine position at full inspiration. Images were contiguously reconstructed with 1 mm slice thickness with 0.625 mm overlap using a standard kernel algorithm. The tube voltage and current were 120 kV and 220 mA, respectively. CT images were automatically analyzed using the FACT-Digital Lung system [28]. A three-dimensional approach was used to measure the percentage of lung volume with a CT attenuation value of <−950 Hounsfield units (HU). A threshold of −950 HU was used to analyze the presence of emphysema by CT densitometry [29], and the EI was quantified using the percentage of LAA based on the lung volume in inspiratory images according to the method previously described [30].

### 2.5. Statistical Analyses

The SPSS 19.0 software (IBM Corporation, Armonk, NY, USA) was used for statistical analyses. Data were expressed as mean ± standard deviation (SD) for continuous variables conforming to a normal distribution or expressed as median (interquartile range) for noncontinuous variables. Categorical variables were expressed as *n* (%). Receiver operating characteristic (ROC) curves were analyzed to evaluate the diagnostic efficacy of the EI for differentiating CB from COPD. Differences between groups were analyzed by one-way analysis of variance followed by Tukey's method, the Kruskal–Wallis test followed by the Bonferroni method, or the Chi-squared test followed by the Bonferroni method. *p* < 0.05 indicated a statistically significant difference.

## 3. Results

### 3.1. Patient Characteristics

**Figure 1:**
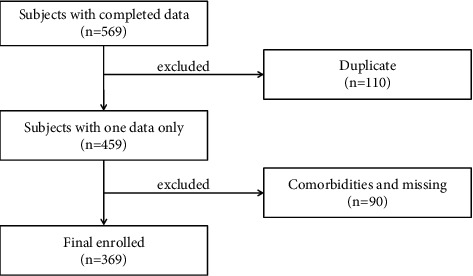
Screening flow chart. A total of 369 patients with complete datasets were enrolled after excluding 110 patients who were rehospitalized and 90 patients who exhibited comorbidities and/or had missing data.

A total of 569 patients were recruited in our study between 2014 and 2017. Based on the inclusion and exclusion criteria, a total of 369 subjects, including 277 COPD patients and 92 CB cases (Figure 1), were enrolled. The baseline characteristics of all patients are presented in Table 1.

### 3.2. Receiver Operating Characteristic (ROC) Curve Analysis

**Figure 2:**
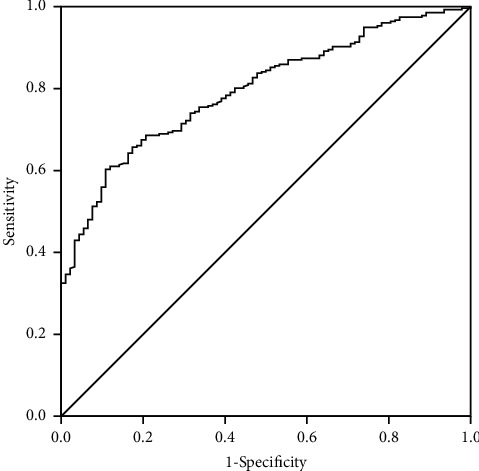
Receiver operating characteristic curve of the emphysema index (EI). The application of the EI to detect chronic obstructive pulmonary disease (COPD) has diagnostic efficacy, and the optimal EI cutoff value (EI = 16.2%) for distinguishing COPD from chronic bronchitis had the highest diagnostic efficacy when the sensitivity was 60.3% and specificity was 89.1%. Youden index = 0.494.

The pulmonary function test is the gold standard for diagnosing COPD. ROC curve analysis showed that the area under the curve (AUC) was 0.796 and an EI of 16.2% (95% confidence interval (CI), 0.750–0.843) was identified as the cutoff value to distinguish COPD from CB (Figure 2). All these suggested that EI was effective in diagnosing COPD.

### 3.3. Pulmonary Function in COPD

The patients with COPD were classified into mild and moderate-to-severe emphysema groups based on the EI; moderate-to-severe emphysema was defined as an EI ≥ 16%. Then, patients were further subdivided based on the CAT score. Finally, patients were divided into Group 1 (EI < 16% and CAT < 10, *n* = 20), Group 2 (EI < 16% and CAT ≥ 10, *n* = 89), Group 3 (EI ≥ 16% and CAT < 10, *n* = 32), and Group 4 (EI ≥ 16% and CAT ≥ 10, *n* = 136). The baseline characteristics of these four groups are listed in Table 2.

**Figure 3:**
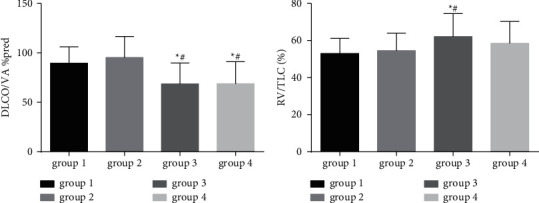
Comparison of DLCO/VA % predicted and RV/TLC among groups. Statistically significant differences were observed between Groups 1 and 2 and Groups 3 and 4 in DLCO/VA % predicted, while the RV/TLC was significantly higher in Group 3 than in Groups 1 and 2. ^*∗*^*p* < 0.05, compared to Group 1; ^#^*p* < 0.05, compared to Group 2. DLCO = carbon monoxide-diffusing capacity, VA = alveolar ventilation, RV = residual volume, and TLC = total lung capacity.

**Figure 4:**
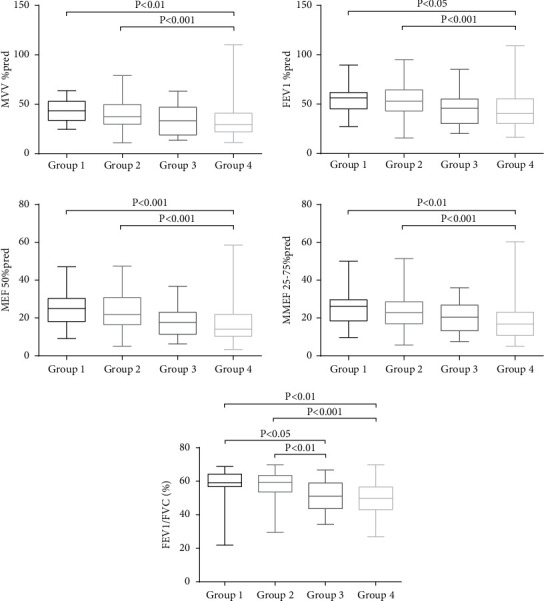
Comparison of pulmonary functions among the four groups. Compared to Group 4, Groups 1 and 2 exhibited statistically significant differences in MVV % predicted, FEV1 % predicted, MEF 50% predicted, and MMEF 25–75% predicted; additionally, significant differences were observed between Groups 1 and 2 and Groups 3 and 4 in FEV1/FVC % predicted. MVV = maximal voluntary ventilation, FEV1 = forced expiratory volume in 1 second, MEF = maximal expiratory flow, MMEF = maximal midexpiratory flow, and FVC = forced vital capacity.

As shown in Figures 3 and 4, significant differences were observed in the MVV % pred (Group 1: 43.4 [33.7, 52.9] and Group 2: 37.4 [29.8, 49.8]), MEF 50% pred (Group 1: 25.0 [18.0, 30.3] and Group 2: 21.8 [16.4, 30.8]), and MMEF 25–75% pred (Group 1: 26.3 [18.4, 29.6] and Group 2: 22.8 [17.0, 28.7]) of Groups 1 and 2, compared to Group 4 (MVV % pred: 29.5 [22.0, 40.9]; MEF 50% pred: 14.1 [10.3, 21.9]; MMEF 25–75% pred: 16.8 [10.8, 23.0]) (all *p* < 0.01). FEV1/FVC (Group 3: 51.1 [43.6, 59.0] and Group 4: 49.8 [43.0, 56.6]) and carbon monoxide-diffusing capacity (DLCO)/alveolar ventilation (VA) %pred (Group 3: 68.7 ± 21.0, Group 4: 68.8 ± 22.2) in Groups 3 and 4 were significantly lower than those in Groups 1 and 2 (all *p* value <0.05). In contrast, the air trapping parameter (RV/TLC) was significantly higher in Group 3 (62.1 ± 12.5) than in Groups 1 (53.0 ± 8.2) and 2 (54.6 ± 9.4) (all *p* < 0.05).

## 4. Discussion

COPD is characterized by airflow limitation and chronic inflammation. In the past, COPD was mainly diagnosed by pulmonary function testing. Although the pulmonary function is essential for COPD diagnosis, compliance and tolerability are relatively low, particularly in the elderly. This leads to overdiagnosis of pulmonary function impairment in COPD patients [31]. A study by Lutchmedial et al. suggested that 10.4% of patients had imaging features of emphysema and COPD-related symptoms, but their pulmonary function did not indicate airflow limitation [32]. Therefore, it is inappropriate to solely use pulmonary function as a diagnostic and assessment criterion for COPD. Identification of a suitable stratified approach to accurately determine COPD subtypes has been a challenge for clinicians.

The development of imaging phenotypes provides a novel direction for the study of COPD. A QCT study of emphysema can accurately reflect pathological changes in COPD [33]. Early emphysematous lesions can be detected by QCT. Currently, emphysema-dominant and airway-dominant are classified as the two phenotypes of COPD according to imaging characteristics [13]. Besides, the CAT score can be beneficial for symptom assessment. Our study aimed to evaluate the role of the EI and CAT score in the assessment of COPD and explore whether EI could distinguish COPD from CB.

EI is recognized as an objective parameter for the evaluation of emphysema [34]. However, there are no specific criteria for EI to evaluate the severity of emphysema [35]. The EI cutoff value of 15% was mostly used in relevant studies [36, 37]. In our study, we found that EI = 16.2% was a cutoff value to distinguish between COPD and CB patients, similar to findings in the previous report mentioned above. Moreover, to confirm this, the analysis in Section 2 was also performed under the EI cutoff of 15%, and similar results were obtained with the results under EI cutoff of 16% (data not shown). Thus, EI of 16% may be the threshold value for evaluating emphysema severity. In our study, approximately 61% of patients with COPD had an EI ≥ 16.2%, who were classified as emphysema-dominant COPD and the remaining subjects were classified as non-emphysema-dominant COPD.

In addition, patients with emphysema-dominant COPD or non-emphysema-dominant COPD were subgrouped according to the CAT score. Our data showed that patients with non-emphysema-dominant COPD in Groups 1 and 2 showed significantly higher MVV % pred, FEV1 % pred, FEV1/FVC, MEF 50% pred, MMEF 25–75% pred, and DLCO/VA % pred than in Group 4. Moreover, the FEV1/FVC, DLCO/VA % pred, and RV/TLC were significantly lower in patients with emphysema-dominant COPD in Group 3, than those in Groups 1 and 2. Conversely, the RV/TLC was significantly higher in Group 3 than in Groups 1 and 2. Thus, the emphysema-dominant patients had a poorer pulmonary function.

The FEV1/FVC and DLCO/VA % pred were higher in patients with mild emphysema with severe symptoms than in those with moderate-to-severe emphysema without severe symptoms, implying that more severe pulmonary parenchyma destruction and airflow obstruction occurred earlier in the emphysema-dominant group. Overall, the emphysema-dominant group exhibited higher RV/TLC, indicating a higher degree of air trapping in emphysema-dominant patients than in non-emphysema-dominant patients. Our results also revealed that the MVV % pred, FEV1 % pred, MEF 50% pred, and MMEF 25–75% pred were lower in the non-emphysema-dominant group with severe symptoms than in the emphysema-dominant group without severe symptoms. Thus, we assumed that patients without apparent imaging changes would benefit from symptom assessment. In addition, we found that it was more convenient to screen non-emphysema-dominant patients with severe symptoms based on the combined EI and CAT score. Furthermore, pulmonary function was not significantly different between non-emphysema-dominant patients with and without severe symptoms, indicating that it was difficult to distinguish Groups 1 and 2 by pulmonary function only.

Our study had some limitations. First, our study was limited by its retrospective design. Second, the sex ratios of the four groups were significantly different, potentially causing bias. Finally, we assessed only the combination of CAT score and EI in COPD evaluation. Combining multiple indicators may increase the accuracy of COPD stratification. Thus, further analysis with a large sample size including combining multiple indicators is urgently needed.

## 5. Conclusion

In summary, the combination of EI and CAT score was effective in distinguishing COPD patients of different phenotypes. The emphysema-dominant patients had a poorer pulmonary function. The non-emphysema-dominant patients benefited from the symptom assessment. However, pulmonary function was not significantly different between non-emphysema-dominant patients with and without severe symptoms. Our findings may be helpful in guiding individualized therapy and the clinical management of different subtypes of COPD. However, studies on novel COPD evaluation systems with multiple indicators are warranted.

## Figures and Tables

**Table 1:** Basic characteristics of the included patients.

Variables	CB (*n* = 92)	COPD (*n* = 277)	*x* ^2^/*t*	*p*
Sex, *n* (%)			7.970	0.005
Male	69 (75.0)	242 (87.4)		
Female	23 (25.0)	35 (12.6)		
Age (years)	70.7 ± 10.0	67.4 ± 10.3	2.671	0.008
COPD education, *n* (%)			0.081	0.777
Yes	35 (38.0)	110 (39.7)		
No	57 (62.0)	167 (60.3)		
BMI (kg/m^2^)	24.2 (22.1, 26.0)	23.7 (20.5, 26.4)	1.285	0.199
Smoking (pack-years)	30.0 (20.0, 40.0)	40.0 (21.9, 50.0)	−2.573	0.010
mMRC, *n* (%)			3.835	0.429
0	28 (30.4)	76 (27.4)		
1	29 (31.5)	84 (30.3)		
2	24 (26.1)	60 (21.7)		
3	9 (9.8)	50 (18.1)		
4	2 (2.2)	7 (2.5)		
WBC (× 10^9^/L)	5.91 (4.73, 6.92)	6.76 (5.43, 8.55)	−4.032	<0.001
N (%)	65.2 (57.3, 73.6)	72.7 (64.3, 79.4)	−4.693	<0.001
E (%)	2.20 (1.00, 3.76)	1.50 (0.50, 3.00)	−2.769	0.006
PaO_2_ (mmHg)	83.0 (71.5, 89.5)	75.0 (65.5, 85.0)	−2.713	0.007
PaCO_2_ (mmHg)	37.0 (35.0, 41.0)	42.0 (37.0, 47.0)	−4.765	<0.001
FEV1 (L)	2.01± 0.65	1.32 ± 0.52	10.336	<0.001
FEV1/FVC (L)	78.4 ± 6.2	53.3 ± 9.8	28.829	<0.001

COPD = chronic obstructive pulmonary disease, CB = chronic bronchitis, BMI = body mass index, mMRC = modified Medical Research Council, WBC = white blood cell count, N = neutrophil count, E = eosinophil count, and Pa = partial pressure.

**Table 2:** Basic information of patients in the four chronic obstructive pulmonary disease groups.

Variables	EI < 16%		EI ≥ 16%		*p* value
	CAT < 10 (Group 1, *n* = 20)	CAT ≥ 10 (Group 2, *n* = 89)	CAT < 10 (Group 3, *n* = 32)	CAT ≥ 10 (Group 4, *n* = 136)	
Sex, *n* (%)					<0.001^a^
Male	14 (70.0)	68 (76.4)	31 (96.93)^#^	129 (94.9)^#,^^*∗*^	
Female	6 (30.0)	21 (23.6)	1 (3.1)	7 (5.1)	
Age (years)	63.4 ± 11.3	67.7 ± 11.0	67.8 ± 11.0	67.7 ± 9.6	0.350^b^
COPD education					0.030^a^
Yes, *n* (%)	13 (65.0)	27 (30.3)^#^	14 (43.8)	56 (41.2)	
No, *n* (%)	7 (35.0)	62 (69.7)	18 (56.2)	80 (58.8)	
BMI (kg/m^2^)	23.8 (21.6,27.3)	25.7 (22.5,27.7)	22.7 (19.5,25.5)^*∗*^	22.2 (19.6,25.1)^*∗*^	<0.001^c^
Smoking (pack-years)	40.0 (32.5,62.5)	36.5 (20.0,50.0)	40.0 (20.0,60.0)	40.0 (25.0,50.0)	0.302^c^
WBC (×10^9^/L)	7.48 (5.20,9.19)	6.71 (5.51,8.24)	7.30 (5.38,10.34)	6.49 (5.24,8.44)	0.587^c^
N (%)	71.56 ± 12.67	71.28 ± 10.44	71.76 ± 15.07	71.70 ± 11.15	0.994^b^
E (%)	0.80 (0.40,3.48)	1.40 (0.35,3.05)	1.60 (0.48,2.90)	1.50 (0.50,3.08)	0.869^c^
PaO_2_ (mmHg)	80.0 (64.5,90.8)	76.0 (66.0,88.0)	75.5 (66.0,82.8)	73.0 (62.5,84.8)	0.628^c^
PaCO_2_ (mmHg)	42.0 (37.0,45.8)	39.0 (35.0,44.0)	42.0 (37.0,46.5)	43.5 (39.0,49.0)^*∗*^	<0.001^c^

COPD = chronic obstructive pulmonary disease, CAT = COPD Assessment Test, EI = emphysema index, BMI = body mass index, WBC = white blood cell count, N = neutrophil count, E = eosinophil count, and Pa = partial pressure. ^a^Chi-squared analysis (Bonferroni method for difference between groups), ^b^analysis of variance (Tukey's method for difference between groups), and ^c^Kruskal–Wallis test (Bonferroni method for difference between groups). #*p* < 0.05, versus Group 1; ^*∗*^*p* < 0.05, versus Group 2.

## Data Availability

All data generated or analyzed during this study are included within this article.

## Abbreviations

EI: Emphysema index
CB: Chronic bronchitis
COPD: Chronic obstructive pulmonary disease
CAT: COPD Assessment Test
ROC: Receiver operating characteristic
MVV: Maximal voluntary ventilation
FVC: Forced vital capacity
FEV1: Forced expiratory volume in 1 second
DLCO: Carbon monoxide-diffusing capacity
VA: Alveolar ventilation
QCT: Quantitative computed tomography
CT: Computerized tomography
LAA: Low attenuation area
TLC: Total lung capacity
RV: Residual volume
MMEF: Maximal midexpiratory flow.
